# Controlled synthesis of bilayer graphene on nickel

**DOI:** 10.1186/1556-276X-7-437

**Published:** 2012-08-06

**Authors:** Ahmad Umair, Hassan Raza

**Affiliations:** 1 Department of Electrical and Computer Engineering, University of Iowa, Iowa City, IA, 52242, USA

**Keywords:** Bilayer graphene, Chemical vapor deposition, Raman spectroscopy

## Abstract

We report a uniform and low-defect synthesis of bilayer graphene on evaporated polycrystalline nickel films. We used atmospheric pressure chemical vapor deposition with ultra-fast substrate cooling after exposure to methane at 1,000°C. The optimized process parameters, i.e., growth time, annealing profile and flow rates of various gases, are reported. By using Raman spectroscopy mapping, the ratio of 2D to G peak intensities (*I*_2D_/*I*_G_) is in the range of 0.9 to 1.6 over 96% of the 200 μm × 200 μm area. Moreover, the average ratio of D to G peak intensities (*I*_D_/*I*_G_) is about 0.1.

## Background

Graphene, a monolayer of sp^2^-hybridized C atoms arranged in a honeycomb structure, has attracted a lot of attention due to its excellent electrical, mechanical and optical properties [1-3]. Monolayer and bilayer graphene (BLG) are semi-metals with zero band gap. The intrinsic band gap has been the key in semiconducting devices. Band gap can be induced by patterning graphene into nano-ribbons [4-10]. Another method to introduce band gap is to apply electric field in the stacking direction of BLG [11-14].

Graphene synthesis on transition metals by chemical vapor deposition (CVD) or via segregation of solid carbon sources is generally a scalable process [15-24]. Transition metals such as Ni, Cu, Pt, Ir and Pd have been used as substrates for graphene growth [25-29]. Several hydrocarbons, like methane (CH_4_), acetylene, ethylene, propane, etc., have been used in atmospheric and low-pressure CVD as a carbon source [25]. Besides the CVD of the above mentioned gases, C_60_ and solid polymers such as poly(methyl metha-crylate), polystyrene and acrylonitrile butadiene styrene have also been decomposed to grow graphene [30-32].

Graphene synthesizes on Ni due to segregation of carbon at high temperatures. Due to the high solubility of carbon in Ni, precipitation of extra carbon occurs at the metal surfaces during cooling. Since the precipitation is a non-equilibrium process, this makes thickness control of graphene a challenge [33,34]. The segregation of extra carbon during the cooling process can be decreased by reducing Ni film thickness. Moreover, extra carbon segregation can also be controlled by controlling the cooling rate [15,22,35-37]. Very high cooling rate has been reported to deposit amorphous carbon deposition, whereas low cooling rate leads to no growth [15]. It has been reported that the carbon segregation on Ni is non-uniform at low temperatures. However, CH_4_ has high decomposition temperature, which helps in constant carbon coverage over the Ni surfaces [38]. Furthermore, high melting point of Ni enables high-temperature annealing, which results in larger domains, thus making it favorable for large-area low-defect growth [25].

In this paper, we report a method to control the precipitation of extra carbon on Ni surface during the cooling-down process. We reduce the sample temperature from the growth temperature of 1,000°C to room temperature in a few seconds, which leads to a uniform BLG growth.

## Methods

BLG was grown on a 300 nm Ni film, evaporated on SiO_2_ (300 nm)/Si substrate. SiO_2_/Si substrate was treated with acetone (10 min), methanol (10 min), deionized (DI) water rinse (10 min) and nanostrip (20 min; commercial Piranha substitute), followed by another DI water rinse (10 min). After cleaning, Ni was evaporated by using an e-beam evaporator at 1Å/s. Ni/SiO_2_/Si samples were gently cleaned in UV ozone for 2 min before loading in the CVD furnace. UV ozone eliminates organic contaminants from the Ni film, which is important for uniform growth. Process gases were supplied by Airgas (Denver, CO, USA) with research grade 5.0 (minimum purity 99.999%). The samples were loaded into the CVD furnace (1-inch tube diameter; Lindbergh/Blue, Thermo Scientific, Logan, UT, USA) at room temperature and heated to 700°C in 200 sccm Ar ambient. At 700°C, 65 sccm H_2_ was introduced in addition to Ar, and the samples were annealed for another 10 min. The temperature was ramped to 1,000°C in Ar/H_2_ ambient. To stabilize the growth temperature, the samples were further annealed for 10 min after reaching 1,000°C. Ar/H_2_ annealing sequence leads to increased grain size and decreased surface roughness [18,22]. Finally, H_2_ was turned off, and BLG was synthesized by introducing CH_4_ into the furnace in addition to the already flowing Ar gas. A wide process parameter space was explored, which includes (a) varying the growth time (50, 60 and 120 s) under a constant CH_4_ flow rate (23 sccm) and (b) varying the flow rates (6, 12 and 23 sccm) under a constant growth time (120 s). After the growth, the sample temperature was reduced to room temperature within a few seconds by pulling the quartz tube out of the hot region of the furnace.

For material characterization, micro-Raman spectroscopy (Raman Nicolet Almega XR Spectrometer, Thermo Scientific) was used in the point scan and the area scan mode [3,39,40]. A 532 nm laser (10 mW power) was used with a 0.6 μm spot size, 15 s scan time and four scans per point. To examine the uniformity of the synthesized graphene, the ratio of 2D to G peak intensities (*I*_2D_/*I*_G_ ratio) was taken over an area of 200 μm × 200 μm. In the area scan, a 2.1 μm spot size was used with 15 s scan time, four scans per point and 10 μm step size.

## Results and discussions

**Figure 1:**
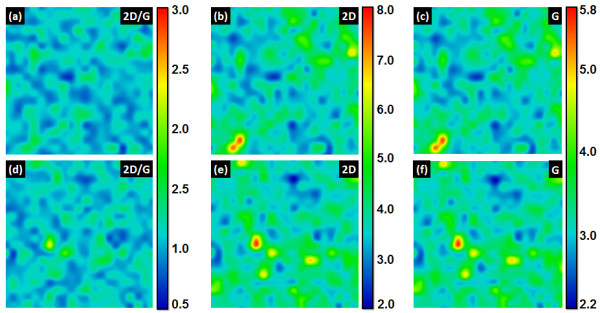
** Two-dimensional Raman intensity map for bilayer graphene.** (**a**) *I*_2D_/*I*_G_ ratio (ratio of 2D to G peak intensities). (**b**) *I*_2D_ (intensity of 2D peak). (**c**) *I*_G_ (intensity of G peak). (**d**), (**e**) and (**f**) show *I*_2D_/*I*_G_, *I*_2D_ and *I*_G_, respectively, for a different area. BLG was grown by using CVD on 300-nm of evaporated Ni film under CH_4_/Ar (23:200 sccm) at 1,000°C for 120 s. The total area of each view map is 200 μm × 200 μm.

Figure 1a,d shows the ratio of 2D to G peak intensities (*I*_2D_/*I*_G_) at two different locations for a sample that was grown under 23 sccm CH_4_ for 120 s. In each case, the *I*_2D_/*I*_G_ ratio is in the range of 0.9 to 1.6 over 96% of the total 200 μm × 200 μm area. This suggests that the BLG is grown over a larger percentage of area on polycrystalline Ni film [21,22,39,40]. The *I*_2D_ and *I*_G_ plots over these locations are shown in Figure 1b,e and Figure 1c,f, respectively. These plots show a uniform intensity distribution for the G and D peaks, which further implies the graphene sample uniformity. 

**Figure 2:**
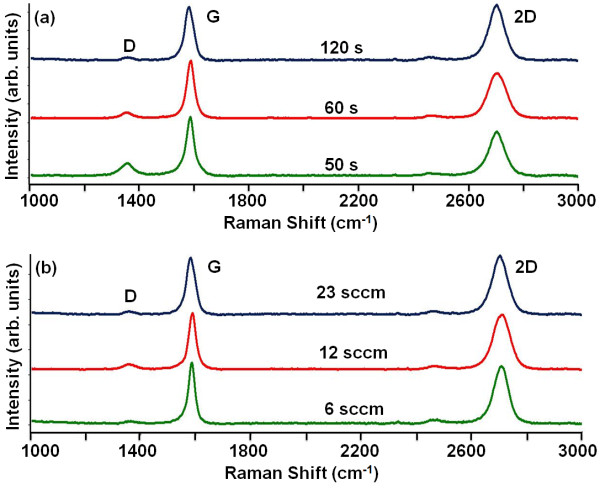
** Raman spectra for various growth conditions.** (**a**) Increasing the growth time decreases the D peak intensity for 23 sccm of CH_4_. (**b**) BLG quality is uniform over wide CH_4_ flow rates for 120-s growth time.

Next, the growth time was varied to study the effect on the number of layers, uniformity and defect density of the synthesized graphene. Figure 2a shows the Raman spectra of the samples that were treated under 23 sccm of CH_4_ for 50, 60 and 120 s. The *I*_2D_/*I*_G_ ratio is close to unity with these varying growth times. This quenching method inhibits the precipitation of extra C on the Ni surface and thus controls the number of layers and the uniformity of the graphene for various growth times. Furthermore, after turning the CH_4_ off, if there is some residual C inside the furnace, the ultra-fast cooling suppresses its further segregation. Moreover, the effect of the CH_4_ flow rate was also studied for constant growth time. Figure 2b shows the Raman spectra of samples for which the growth time was 120 s with CH_4_ flow rates of 6, 12 and 23 sccm. It shows that the BLG growth is consistent for a wide range of flow rates.

**Figure 3:**
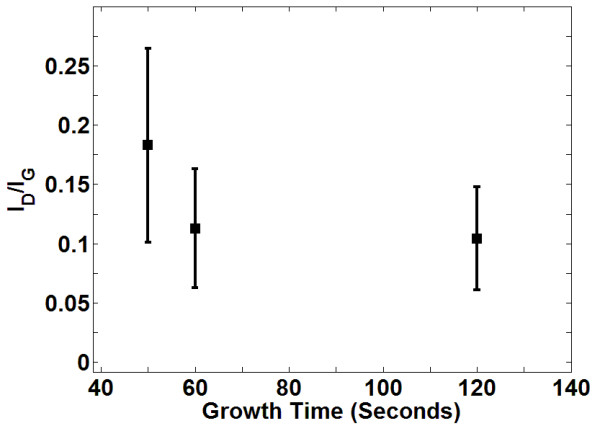
*** I***_**D**_**/*I***_**G**_**ratio (ratio of D to G peak intensities).** The average defect density decreases with increasing growth time.

Another important observation is that the intensity of the D peak decreases as the growth time increases from 50 to 120 s, as shown in Figure 2a. The ratio of D to G peak intensities (*I*_D_/*I*_G_ ratio) was taken over 20 locations for the samples grown under 23 sccm of CH_4_ for 50, 60 and 120 s. The mean and standard deviation of *I*_D_/*I*_G_ ratio are plotted in the error bar graph shown in Figure 3. The average *I*_D_/*I*_G_ ratio for the sample grown under 23 sccm of CH_4_ for 120 s is 0.1 with a standard deviation of 0.05, which suggests a low defect density of BLG for these parameters. Moreover, Figure 3 also shows that the average defect density decreases with the increasing growth time. Figure 2b also shows that the defect density of BLG is small for the samples grown under wide CH_4_ flow rates for 120 s.

We find that quenching the samples from the hot region of the furnace helps in reducing the non-equilibrium precipitation of extra carbon on the Ni surfaces during the cooling process, and that the main growth mechanism is diffusion of carbon on Ni surface due to the decomposed CH_4_. With fast cooling, the reduced sample temperature stops further segregation of carbon due to any residual carbon inside the furnace, even after CH_4_ flow was turned off. The thickness of the graphene is almost constant even with a wide range of CH_4_ flow rates (6 to 23 sccm), which shows that the segregation process is rather self-limiting. Furthermore, the growth temperature is high due to high decomposition temperature of CH_4_ that supports the uniform carbon diffusion over the Ni surface. This helps in growing uniform BLG with less defect density. Moreover, as the growth time is decreased, the average intensity of the D peak increases, which indicates incomplete growth. This further verifies the self-limiting equilibrium segregation of carbon on Ni surface, with reduced out-diffused carbon atoms from the C-Ni solution due to fast cooling. To verify the proposed growth mechanism, graphene was grown on 300 nm Ni film, with 23 sccm CH_4_ flow rated for 120 s, cooling the samples within the furnace. Due to slow cooling, the precipitation of carbon on Ni surface from the C-Ni solution is a dominant process.

Yet, another way to reduce the precipitation of extra carbon is to reduce the thickness of Ni film as less thick films would absorb less carbon and thus contribute to further decrease in out-diffused carbon. To characterize this effect, the growth was performed on 200 and 100 nm thick Ni films, with 23 sccm CH_4_ flow rate for 120 s. For the 200 nm Ni film, the *I*_2D_/*I*_G_ ratio is close to unity, and the area uniformity is similar to the 300 nm thick films. However, growth on 100 nm Ni film results in increased surface roughness. Although the *I*_2D_/*I*_G_ ratio is still around unity in this process, surface coverage is only 50%.

## Conclusions

In conclusion, we have reported a method to synthesize bilayer graphene through CVD of CH_4_ on polycrystalline Ni films with an ultra-fast cooling technique. The number of graphene layers is uniform over a wide area with low defect density. The growth is consistent over a certain variation of CH_4_ flow rate and growth time.

## Competing interests

Both authors declare that they have no competing interests.

## Authors’ contribution

HR and AU have equal contribution to this work and the manuscript. Both authors read and approved the final manuscript.
